# Ultrasound-Enhanced Catalytic Ozonation Oxidation of Ammonia in Aqueous Solution

**DOI:** 10.3390/ijerph16122139

**Published:** 2019-06-17

**Authors:** Chen Liu, Yunnen Chen, Caiqing He, Ruoyu Yin, Jun Liu, Tingsheng Qiu

**Affiliations:** Jiangxi Key Laboratory of Mining & Metallurgy Environmental Pollution Control, Jiangxi University of Science & Technology, Ganzhou 341000, China; chen_liu94@163.com (C.L.); m17750651450@163.com (C.H.); yry9527@163.com (R.Y.); Liuj1979@163.com (J.L.); qiutingsheng@163.com (T.Q.)

**Keywords:** ammonia, ultrasound (US), catalytic ozonation, SrO-Al_2_O_3_ catalyst

## Abstract

Excessive ammonia is a common pollutant in the wastewater, which can cause eutrophication, poison aquatic life, reduce water quality and even threaten human health. Ammonia in aqueous solution was converted using various systems, i.e., ozonation (O_3_), ultrasound (US), catalyst (SrO-Al_2_O_3_), ultrasonic ozonation (US/O_3_), ultrasound-enhanced SrO-Al_2_O_3_ (SrO-Al_2_O_3_/US), SrO-Al_2_O_3_ ozonation (SrO-Al_2_O_3_/O_3_) and ultrasound-enhanced SrO-Al_2_O_3_ ozonation (SrO-Al_2_O_3_/US/O_3_) under the same experimental conditions. The results indicated that the combined SrO-Al_2_O_3_/US/O_3_ process achieved the highest NH_4_^+^ conversion rate due to the synergistic effect between US, SrO-Al_2_O_3_ and O_3_. Additionally, the effect of different operational parameters on ammonia oxidation in SrO-Al_2_O_3_/O_3_ and SrO-Al_2_O_3_/US/O_3_ systems was evaluated. It was found that the ammonia conversion increased with the increase of pH value in both systems. The NH_3_(aq) is oxidized by both O_3_ and ·OH at high pH, whereas the NH_4_^+^ oxidation is only carried out through ·OH at low pH. Compared with the SrO-Al_2_O_3_/O_3_ system, the ammonia conversion was significantly increased, the reaction time was shortened, and the consumption of catalyst dosage and ozone were reduced in the SrO-Al_2_O_3_/US/O_3_ system. Moreover, reasonable control of ultrasonic power and duty cycle can further improve the ammonia conversion rate. Under the optimal conditions, the ammonia conversion and gaseous nitrogen yield reached 83.2% and 51.8%, respectively. The presence of *tert*-butanol, CO_3_^2−^, HCO_3_^−^, and SO_4_^2−^ inhibited the ammonia oxidation in the SrO-Al_2_O_3_/US/O_3_ system. During ammonia conversion, SrO-Al_2_O_3_ catalyst not only has a certain adsorption effect on NH_4_^+^ but accelerates the O_3_ decomposition to ·OH.

## 1. Introduction

Ammonia is a common contaminant. In particular, a large amount of ammonia wastewater is produced in the process of mining extraction and separation of rare-earth ore [1]. Once the discharge of ammonia exceeds the environmental capacity of the receiving waters, it causes several problems, including eutrophication, poisoning aquatic life, reducing water quality, and even threatening human health [2,3]. Hence, it is necessary to treat the ammonia in wastewater.

As an oxidant, ozone (O_3_) has been used in wastewater treatment and deep purification of drinking water [4,5]. Ozonation alone has low oxidation efficiency because of its unstable chemical properties (the half-life is approximately 15 min under neutral conditions at 298 K), which limits its oxidation ability [6,7]. To improve the oxidation efficiency of ozone, the synergy of catalyst has been investigated, namely catalytic ozonation, which can be classified into homogeneous and heterogeneous depending on the type of catalyst [8,9]. In homogeneous catalytic ozonation, liquid catalysts (mostly transition metal ions) are used to decompose the ozone, but it is difficult to separate from the effluent, thus causing secondary pollution in water bodies [9,10]. In heterogeneous catalytic ozonation, solid catalysts that are easily separated and maintain their catalytic activity for a long time are employed for decomposing ozone into hydroxyl radicals (·OH) [9,11]. ·OH is more oxidative than O_3_, because their oxidation potentials are 2.80 V and 2.07 V, respectively [12]. In addition, the solid catalyst in ozonation can directly convert ammonia into harmless substances (such as N_2_) under certain conditions [13,14]. Therefore, the suitable solid catalysts are very important for the ammonia conversion by heterogeneous catalytic ozonation.

Recently, the focus has been on alkaline earth metal oxides due to their lower solubility in the reaction process and the basic oxygen-containing groups (hydroxyl groups) on their surface [15]. The order of catalytic activity of alkaline earth metal oxides per unit surface area is SrO > CaO > MgO [16]. Furthermore, surface hydroxyl groups on the catalyst react easily with O_3_ to form ·OH [12]. However, the alkaline earth metal oxides are powdery, without mechanical strength, and are not easily separated from the effluent [17,18]. To overcome these limitations, attaching the alkaline earth metal oxide to some supports has been considered. Activated alumina, with acid-base active sites on their surface, high mechanical strength and good thermal stability, can be used as a suitable support [18]. Cotman et al. [19] prepared ruthenium metal supported on alumina catalyst (Ru-Al_2_O_3_) to catalyze ozonation of bisphenol A, and the catalyst Ru-Al_2_O_3_ showed excellent stability and reusability. Wang et al. [20] studied the manganese metal loading of 4% on Al_2_O_3_ (Mn-Al_2_O_3_) exhibited highest catalytic activity.

In the process of heterogeneous catalytic ozonation, catalyst deactivation is also an important issue affecting the catalytic efficiency due to the accumulation of by-products on the catalyst surface [21]. Currently, ultrasound (US) is combined with the heterogeneous catalytic ozonation process to treat organic pollutants in water [22,23,24]. When the ultrasound catalytic reaction is carried out, transient cavitation enhances the turbulent of the solution, thereby accelerating the mass transfer process of the reactants and by-products between the solution and the catalyst surfaces [25]. At the same time, the collapse of acoustic cavitation bubbles generates shock waves, thus creating a continuous cleaning effect on the surface of the catalyst [25,26,27]. To date, ultrasound-enhanced catalytic ozonation of ammonia in aqueous solution has rarely been studied.

Accordingly, (1) to enhance the ozone oxidation ability and its utilization rate, (2) to power the mechanical strength of alkaline earth metal oxides and easily separate from the solution, and (3) to delay the deactivation of the catalyst, this study combines an ultrasonic, supported alkaline earth metal oxide with ozone (SrO-Al_2_O_3_/US/O_3_) to oxidize the ammonia in aqueous solution. The effects of initial solution pH, reaction time, dosage of catalyst, ozone flow, ultrasonic power and duty cycle on ammonia conversion were investigated in the SrO-Al_2_O_3_/US/O_3_ system. For comparison, the effects of the same operating parameters on ammonia conversion were studied in SrO-Al_2_O_3_/O_3_ system to understand the role of US in the catalytic ozonation reaction. A set of experiments were also designed for assessing the effect of *tert*-butanol and inorganic ions (Na^+^; K^+^; Ca^2+^; Mg^2+^; SO_4_^2−^; CO_3_^2−^; HCO_3_^−^) on the conversion of ammonia in the SrO-Al_2_O_3_/US/O_3_ system. In addition, the mechanism for ultrasound-enhanced catalytic ozonation of ammonia in aqueous solution is discussed.

## 2. Experimental 

### 2.1. Materials

Ammonium chloride (NH_4_Cl, analytical grade) was purchased from Tianjin Damao Chemical Reagent Factory (Tianjin, China), which was used to prepare simulated water containing NH_4_^+^. Both activated-alumina (Al_2_O_3_, analytical grade) and barium salt (Sr(NO_3_)_2_, analytical grade) were purchased from Shanghai Zhanyun Chemical Co. Ltd. (Shanghai, China).

### 2.2. Preparation of Supported Catalyst

Actived-Al_2_O_3_ (48–212 μm) was washed three times with deionized water, followed by ultrasonic cleaning for 15 min in an ultrasonic cleaner (KQ-100TDE, Kunshan Ultrasonic Instrument Co. Ltd., Kunshan, China) and dried at 110 °C for 5 h in an oven (101A-3, Shanghai Experimental Instrument Factory, Shanghai, China) to obtain pretreated activated-Al_2_O_3_ used as a support for the catalyst.

An impregnation method was used to prepare the SrO-Al_2_O_3_ catalyst. Briefly, the pretreated activated-Al_2_O_3_ and 0.1 mol/L Sr(NO_3_)_2_ solution were mixed in a solid-liquid ratio of 1 g/20 mL and shaken in a 60 °C water bath thermostat (SHA-C, Jintan Ronghua Instrument Manufacturing Co. Ltd., Jintan, China) for 20 h. The mixture was filtrated, dried at 80 °C for 3 h and heated to 110 °C for 2 h in an oven and then calcined at 700 °C for 4 h in a muffle furnace (SHF.M25/10, Shanghai Lingyi Industrial Co. Ltd., Shanghai, China).

### 2.3. Procedure

**Figure 1:**
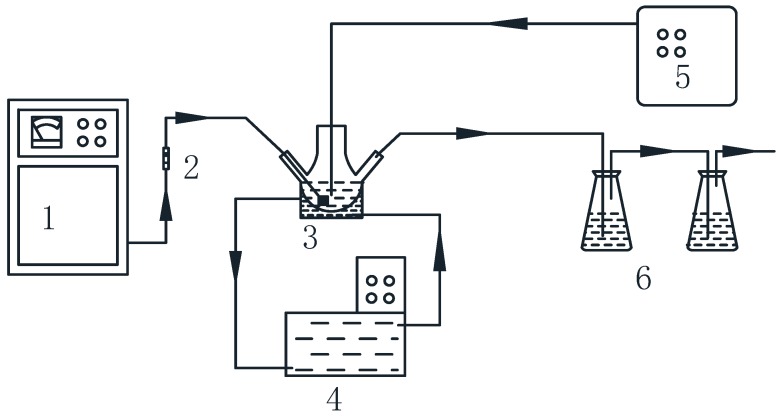
Schematic of experimental apparatus. 1 Ozone generator, 2 Gas-flowmeter, 3 Reactor, 4 Intelligent cryostat, 5 Ultrasonic generator, 6 KI absorption liquid.

The experimental setup of the ultrasound-enhanced catalytic ozonation of ammonia process (SrO-Al_2_O_3_/US/O_3_) is shown in Figure 1. To begin with, 100 mL NH_4_Cl solution was prepared at an initial concentration of 50 mg/L and acidified or alkalized to a set pH value (4.5~11.5) with 1 mol/L HCl or NaOH before each experimental run. Then the resulting solution was placed in a 200 mL reactor and a predetermined amount of SrO-Al_2_O_3_ (0.5~3.0 g/L) was added. Subsequently the instruments were connected and run according to Figure 1. 

O_3_ was generated by an ozone generator (FL-815ET, FeiLi, Shengzhen, China) with an oxygen source and a control device for ozone flow. The ozone generator has a maximum ozone flow of 15 g/h and the ozone flow (0.4~3.8 g/h) was set by the control device. The ozone was continuously bubbled into NH_4_Cl solution and the remaining ozone was absorbed by KI solution. 

Ultrasonic treatment was performed using an ultrasonic generator (XO-SM50N, Nanjing Xianou Instrument Manufacturing Co. Ltd., Nanjing, China) equipped with a 6 mm diameter titanium probe. The probe was inserted to a depth of about 10 mm in the solution and the ultrasound was performed in a pulse mode of 1 s (or 2 s) on and 1 s (or 2 s) off at a given ultrasonic power (90~450 W) and an ultrasonic frequency of 25 Hz. The temperature of the whole reaction system was maintained at 25 °C by an intelligent thermostat.

During the catalytic ozonation of ammonia experiment (SrO-Al_2_O_3_/O_3_), the ultrasonic generator was turned off while other conditions are consistent with SrO-Al_2_O_3_/US/O_3_.

### 2.4. Analysis

After the reaction, liquid samples were withdrawn from the reactor at pre-determined intervals and then the concentrations of ammonium (NH_4_^+^), nitrite (NO_2_^−^), and nitrate (NO_3_^−^) in solution were measured. Nessler’s reagent spectrophotometry method [28] was used for determining the concentration of NH_4_^+^ ($C_{NH_{4}^{+}}$) and the spectrophotometry method [29] for determining the concentration of NO_2_^−^ ($C_{NO_{2}^{-}}$) in the liquid samples by a visible spectrophotometer (SP-756PC, Shanghai Spectrum Instrument Co. Ltd., Shanghai, China). Ultraviolet spectrophotometry method [30] was used for determining the nitrate ($C_{NO_{3}^{-}}$) concentration using an ultraviolet spectrophotometer (722 N, Shanghai Spectrum Instrument Co. Ltd., Shanghai, China). In this study, according to the calculation of nitrogen balance, the products of oxidation of NH_4_^+^ were gaseous nitrogen in addition to residual NH_4_^+^, NO_2_^−^, and NO_3_^−^ in liquid phase. Gaseous nitrogen may include N_2_, N_2_O, NO, NO_2_, etc. [13]. Percentages of NH_4_^+^ ($P_{NH_{4}^{+}}$), NO_3_^−^ ($P_{NO_{3}^{-}}$), NO_2_^−^ ($P_{NO_{2}^{-}}$) and gaseous nitrogen ($P_{\mathrm{gaseous}\;\mathrm{nitrogen}}$) were calculated using Equations (1)–(4):

(1) $$P_{NH_{4}^{+}}=\frac{C_{1}}{C_{0}}\times 100\%$$

(2) $$P_{NO_{3}^{-}}=\frac{C_{2}}{C_{0}}\times 100\%$$

(3) $$P_{NO_{2}^{-}}=\frac{C_{3}}{C_{0}}\times 100\%$$

(4) $$P_{gaseous\;nitrogen}=100\%-P_{NH_{4}^{+}}-P_{NO_{3}^{-}}-P_{NO_{2}^{-}}$$

(5) $$NH_{4}^{+}conversion=100\%-P_{NH_{4}^{+}}$$

where $C_{1}$, $C_{2}$, $C_{3}$ are the concentration, of residual NH_4_^+^, formed NO_3_^−^ and NO_2_^−^ after the reaction, respectively, and $C_{0}$ is the initial ammonia concentration (mg/L).

## 3. Results and Discussions

### 3.1. Performance Comparison of Different Treatment Systems

**Figure 2:**
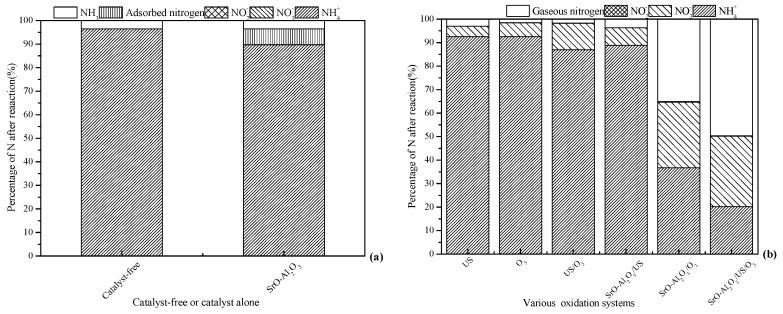
Performance comparison in the various treatment systems on ammonia conversion. Reaction conditions: initial NH_4_^+^ concentration 50 mg/L, initial pH 8.5, ozone flow 1.5 g/h, catalytic dosage 2.0 g/L, reaction temperature 25 °C, reaction time 60 min, ultrasonic frequency 25 kHz, ultrasonic power 270 W, ultrasonic operation 1 s and interval 2 s. (**a**) Catalyst-free or catalyst alone; (**b**) various oxidation systems.

Tests were carried out in the absence and presence of SrO-Al_2_O_3_ to evaluate the adsorption capacity of SrO-Al_2_O_3_ on ammonia, the results is presented in Figure 2a. As shown in Figure 2a, when aqueous solution pH 8.5, 3.5% of NH_4_^+^ was converted to NH_3_ when no catalyst was present. In the SrO-Al_2_O_3_ alone system, the conversion rate of NH_4_^+^ was 10.3% and no nitrate and nitrite nitrogen were produced, since the yield of NH_3_ was 3.5%, indicating that 6.8% of NH_4_^+^ was adsorbed by the SrO-Al_2_O_3_ catalyst.

To evaluate the ammonia conversion rate in various systems, experiments were carried out with US, O_3_, US/O_3_, SrO-Al_2_O_3_/US, SrO-Al_2_O_3_/O_3_ and SrO-Al_2_O_3_/US/O_3_ systems, respectively, and the results are given in Figure 2b. Figure 2b shows that the US alone and O_3_ alone system have a small effect on the oxidation of ammonia in aqueous solution, with ammonia conversion and gaseous nitrogen yield of less than 7.4% and 3.0%, respectively. Simultaneously, the yields of NO_3_^−^ were 4.4% and 5.8%, respectively.

In the US/O_3_ system, less than 13% of NH_4_^+^ was oxidized and the NO_3_^−^ yield was 11.2%, which were higher than those of O_3_ alone and US alone. When US was introduced into the O_3_ system, the propagation of ultrasonic waves in solution produced transient cavitation, causing turbulence and reducing the liquid film thickness of the ozone-containing gas bubbles, which facilitates the mass transfer of ozone [31]. On the other hand, the decomposition of ozone and H_2_O would produce ·OH in the cavitation bubbles (Equations (6)–(8)) [32]. Where the “)))” indicate ultrasound. Subsequently, the cavitation bubbles are ruptured, and the free radicals formed diffuse from the internal into the solution to oxidize the ammonia in aqueous solution [32,33].

(6) $$O_{3}+)))\to \cdot O+O_{2}$$

(7) $$\cdot {O+H}_{2}O\to 2\cdot \mathrm{OH}$$

(8) $$H_{2}O+)))\to \cdot \mathrm{OH}+\cdot H$$

When US was introduced into the SrO-Al_2_O_3_ system, both the ammonia conversion and gaseous nitrogen yield were low (11.2% and 3.6%, respectively). In the SrO-Al_2_O_3_/O_3_ system, the ammonia conversion and production of gaseous nitrogen were 63.2% and 35.1%, respectively. One possible reason for this is that the active sites of SrO-Al_2_O_3_ can stimulate the decomposition of ozone to produce ·OH. Compared to the SrO-Al_2_O_3_/US system, the oxidation effect of ammonia is obviously enhanced, which can be explained by co-oxidation of O_3_ and ·OH in the SrO-Al_2_O_3_/O_3_ system.

As for the SrO-Al_2_O_3_/US/O_3_ system, both ammonia conversion and gaseous nitrogen yield were higher than that of SrO-Al_2_O_3_/O_3_ processes. The reason may be that the active sites of SrO-Al_2_O_3_ were gradually occupied by the NH_4_^+^ and NO_3_^−^ during the catalytic ozonation process in the SrO-Al_2_O_3_/O_3_ system. The active sites of SrO-Al_2_O_3_ were opened by transient cavitation when US was introduced in the SrO-Al_2_O_3_/US/O_3_ system. The experimental results indicated that the ammonia conversion is dominated by the oxidation of O_3_ and ·OH, which comes from the synergistic effect of US, SrO-Al_2_O_3_ and O_3_ in the SrO-Al_2_O_3_/US/O_3_ system.

Comparison with the production of gaseous nitrogen, being 3.5% for the catalyst-free system and 6.8% NH_4_^+^ adsorbed by the SrO-Al_2_O_3_ catalyst in the catalyst alone system in Figure 2a, the NO_3_^−^ can be considered to come from the simultaneous oxidation of NH_4_^+^ and NH_3_ in the O_3_, US, US/O_3_, SrO-Al_2_O_3_/US, SrO-Al_2_O_3_/O_3_ and SrO-Al_2_O_3_/US/O_3_ systems.

### 3.2. Effect of Operating Parameters on Ammonia Conversion

#### 3.2.1. Initial Solution pH 

**Figure 3:**
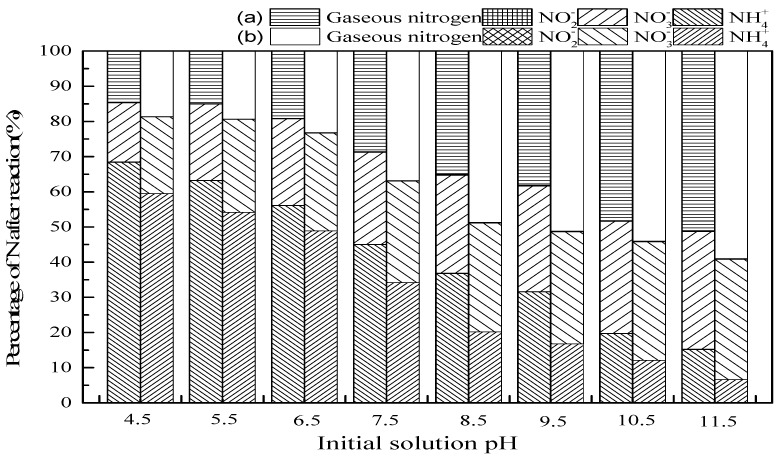
The effect of initial solution pH on ammonia conversion. Reaction conditions: initial NH_4_^+^ concentration 50 mg/L, initial pH 4.5 to 11.5, ozone flow 1.5 g/h, catalytic dosage 2.0 g/L, temperature 25 °C reaction time 60 min, ultrasonic frequency 25 kHz, ultrasonic power 270 W, ultrasonic operation 1 s and interval 2 s. (**a**) SrO-Al_2_O_3_/O_3_, (**b**) SrO-Al_2_O_3_/US/O_3._

Figure 3 depicts the results showing that the pH of the initial solution is positively correlated with ammonia conversion and gaseous nitrogen selectivity. With the initial pH increasing from 4.5 to 11.5, the percentage of NH_4_^+^ decreased, and the production of NO_3_^−^ and gaseous nitrogen increased in the SrO-Al_2_O_3_/O_3_ and SrO-Al_2_O_3_/US/O_3_ system. On the one hand, the form of ammonia in aqueous solution depends on pH (Equation (9)), NH_4_^+^ is predominant at pH lower than pK_a_, and free ammonia (NH_3_(aq)) increases at pH higher than pK_a_ [34].

(9) $${\mathrm{NH}}_{3}(aq)+H_{2}O⇌NH_{4}^{+}+OH^{-};\;{\mathrm{pK}}_{a}=9.246$$

(10) $$\frac{C_{NH_{3}}}{C_{NH_{3}}+C_{NH_{4}^{+}}}=\frac{10^{pH\text{-}14}}{K_{b}+10^{pH\text{-}14}}$$

The fraction of NH_3_ (aq) at a given pH can be calculated according to Equation (10), where K_b_ is the ionization constant, and its value is 1.774 × 10^−5^ at 298 K [3]. Thus, NH_4_^+^ is gradually turned into NH_3_ (aq) with the increase of initial solution pH. For instance, the fraction of NH_3_ (aq) at pH = 4.5 is only 1.8 × 10^−5^, which increases to 0.99 at pH = 11.5.

On the other hand, as shown in Equations (11) and (12), ·OH would be generated from the ozone dissociation at alkaline conditions which contributed to the oxidation NH_3_(aq) [33]. Hence, according to Figure 3, the oxidation of NH_3_(aq) occurs by both O_3_ and ·OH at high pH. As more NH_3_(aq) is continuously oxidized, the reaction equilibrium (9) shifts to the formation of NH_3_(aq), resulting in an increase of ammonia conversion rate. However, since O_3_ cannot oxidize NH_4_^+^ directly [3,32,34], the oxidation of NH_4_^+^ can be carried out through ·OH at low pH, which was derived from ultrasound-enhanced decomposition of O_3_ and H_2_O (Equations (6)–(8)).

(11) $$O_{3}{+\mathrm{OH}}^{-}\to {\mathrm{HO}}_{2}^{-}{+O}_{2}$$

(12) $$O_{3}{+\mathrm{HO}}_{2}^{-}\to \cdot \mathrm{OH}+\cdot O_{2}^{-}{+O}_{2}$$

Comparing the two system SrO-Al_2_O_3_/O_3_ and SrO-Al_2_O_3_/US/O_3_, when the initial pH was 9.5, the introduction of ultrasound increased the ammonia conversion from 68.4% to 83.2%, and the proportion of gaseous nitrogen increased from 38.2% to 51.2%. It is confirmed that the combination of US, SrO-Al_2_O_3_ and O_3_ can synergistically enhance the ammonia conversion.

#### 3.2.2. Reaction Time 

**Figure 4:**
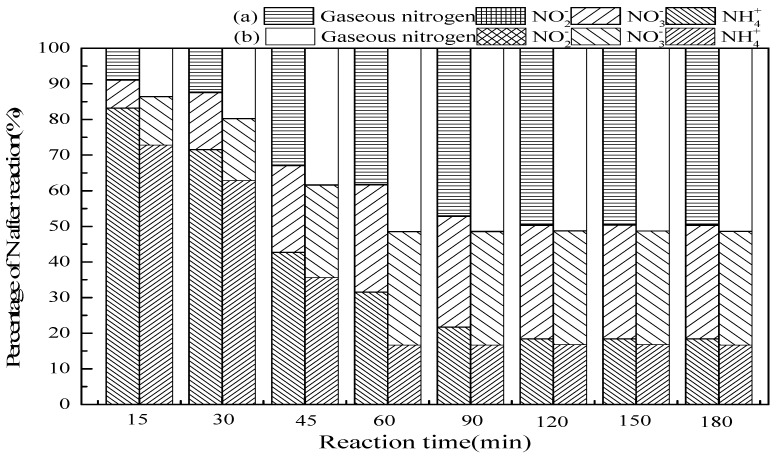
The effect of reaction time on ammonia conversion. Reaction conditions: initial NH_4_^+^ concentration 50 mg/L, initial pH 9.5, ozone flow 1.5 g/h, catalytic dosage 2.0 g/L, temperature 25 °C, reaction time from 15 to 180 min, ultrasonic frequency 25 kHz, ultrasonic power 270 W, ultrasonic operation 1 s and interval 2 s. (**a**) SrO-Al_2_O_3_/O_3_, (**b**) SrO-Al_2_O_3_/US/O_3._

Figure 4 shows that the effect of reaction time on ammonia conversion in SrO-Al_2_O_3_/O_3_ and SrO-Al_2_O_3_/US/O_3_ system. According to Figure 4, the ammonia conversion and the gaseous nitrogen yield increased with the prolongation of the reaction time in the two systems. The reaction time was 120 min in the SrO-Al_2_O_3_/O_3_ system, the ammonia conversion and gaseous nitrogen yield reached 81.6% and 49.6%, respectively, while the reaction time was 60 min in the SrO-Al_2_O_3_/US/O_3_ system, the ammonia conversion and gaseous nitrogen yield reached 83.4% and 51.4%, respectively. In SrO-Al_2_O_3_/O_3_ system, the reaction time for reaching the maximum ammonia conversion rate and gaseous nitrogen yield was twice that of the SrO-Al_2_O_3_/US/O_3_ system, indicating that the introduction of US can greatly shorten the reaction time.

#### 3.2.3. Catalyst Dosage 

**Figure 5:**
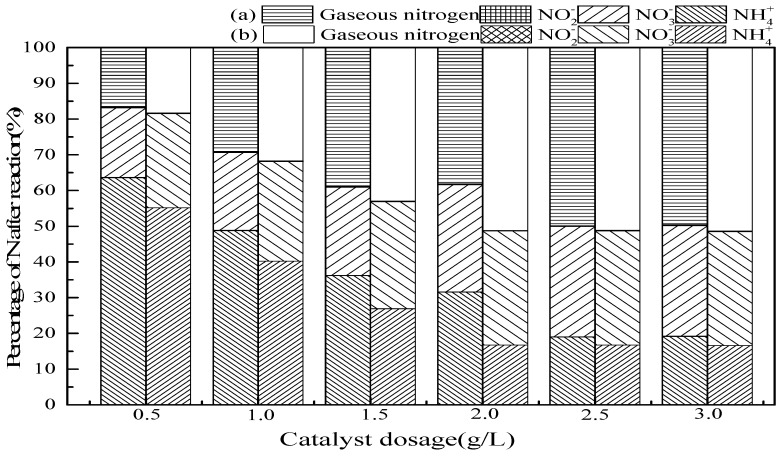
The effect of catalytic dosage on ammonia conversion. Reaction conditions: initial NH_4_^+^ concentration 50 mg/L, initial pH 9.5, ozone flow 1.5 g/h, catalytic dosage from 0.5 g/L to 3.0 g/L, temperature 25 °C, reaction time 60 min, ultrasonic frequency 25 kHz, ultrasonic power 270 W, ultrasonic operation 1 s and interval 2 s. (**a**) SrO-Al_2_O_3_/O_3_, (**b**) SrO-Al_2_O_3_/US/O_3._

In general, increased catalyst dosages provide more surface-active sites, thus facilitating O_3_ decomposition into ·OH [35]. As seen in Figure 5, the ammonia conversion and gaseous nitrogen production improved with an increase of SrO-Al_2_O_3_ dosage. The dosage of SrO-Al_2_O_3_ was 2.5 g/L in the SrO-Al_2_O_3_/O_3_ system, the ammonia conversion and gaseous nitrogen yield reached 81.0% and 50.0%, respectively, while the dosage was 2.0 g/L in the SrO-Al_2_O_3_/US/O_3_ system, the ammonia conversion and gaseous nitrogen yield reached 83.2% and 51.2%, respectively. It is illustrated that the introduction of US cannot only enhance the ammonia conversion but reduce the catalyst dosage.

#### 3.2.4. Ozone Flow

**Figure 6:**
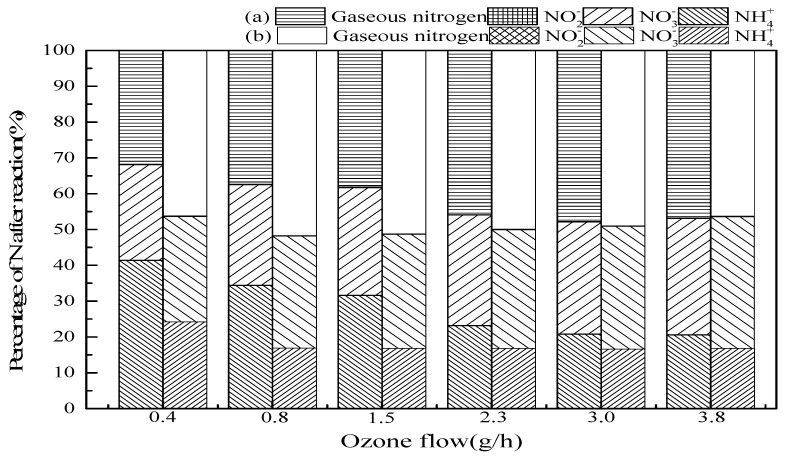
The effect of ozone flow on ammonia conversion. Reaction conditions: initial NH_4_^+^ concentration 50 mg/L, initial pH 9.5, ozone flow from 0.4 to 3.8 g/h, catalytic dosage 2.0 g/L, temperature 25 °C, reaction time 60 min, ultrasonic frequency 25 kHz, ultrasonic power 270 W, ultrasonic operation 1 s and interval 2 s. (**a**) SrO-Al_2_O_3_/O_3_, (**b**) SrO-Al_2_O_3_/US/O_3._

Figure 6 shows the effect of the ozone flow on ammonia conversion. The results indicated that when ozone flow increased from 0.4 mg/min to 3.0 mg/min in SrO-Al_2_O_3_/O_3_ system, the ammonia conversion and gaseous nitrogen yield increased from 58.6% to 79.2% and 31.8% to 47.8%, respectively. While in the SrO-Al_2_O_3_/US/O_3_ system, ozone flow from 0.4 mg/min to 0.8 mg/min, the ammonia conversion and gaseous nitrogen yield increased from 75.8% to 83.1% and 46.2% to 51.7%, respectively. The increase in ozone flow means an increase in ozone concentration, which activates the ·OH generation and ultimately enhances the ammonia oxidation in the reaction system [36]. While continuing to increase the ozone flow, it was found that the ammonia conversion, gaseous nitrogen yield and NO_3_^−^ production in the solution have a little bit increase. The reason for this is that with a certain amount of SrO-Al_2_O_3_ catalyst, the number of active sites is fixed, and cannot completely decompose excessive ozone to ·OH. Comparing the two system of SrO-Al_2_O_3_/O_3_ and SrO-Al_2_O_3_/US/O_3_, it can be concluded that the introduction of US can reduce the consumption of ozone to a large extent.

#### 3.2.5. Ultrasonic Power

**Figure 7:**
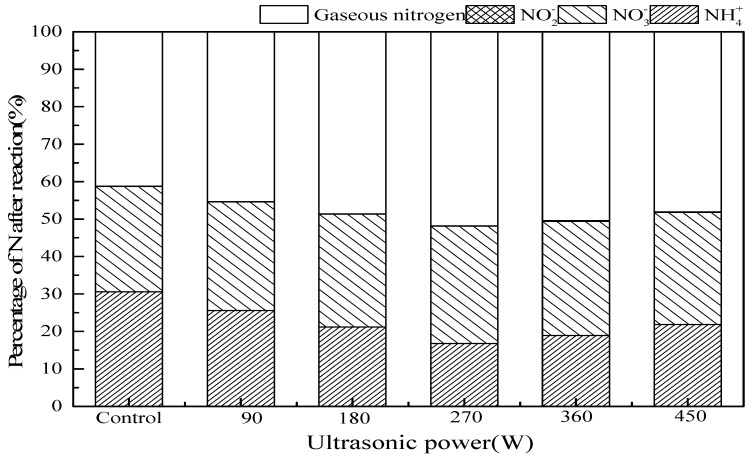
The effect of ultrasonic power on ammonia conversion. Reaction conditions: initial NH_4_^+^ concentration 50 mg/L, initial pH 9.5, ozone flow 0.8 g/h, catalytic dosage 2.0 g/L, temperature 25 °C, reaction time 60 min, ultrasonic frequency 25 kHz, ultrasonic power from 0 to 450 W, ultrasonic operation 1 s and interval 2 s.

Figure 7 shows the effect of ultrasonic power on ammonia conversion in SrO-Al_2_O_3_/US/O_3_ system. Presence of US improved the ammonia oxidation. With the increase of ultrasonic power from 0 to 450 W, residual NH_4_^+^ concentration and gaseous nitrogen yield after the reaction decrease first and then rises. Increasing ultrasonic power from 0 to 270 W can enhance the ultrasonic cavitation, but exceeding 270 W, acoustic shielding appears to reduce the ultrasonic cavitation and the utilization of acoustic energy [31,37]. When the ultrasonic power is 270 W, the ammonia conversion and gaseous nitrogen yield both maximize 83.2% and 51.8%.

#### 3.2.6. Duty Cycle 

**Figure 8:**
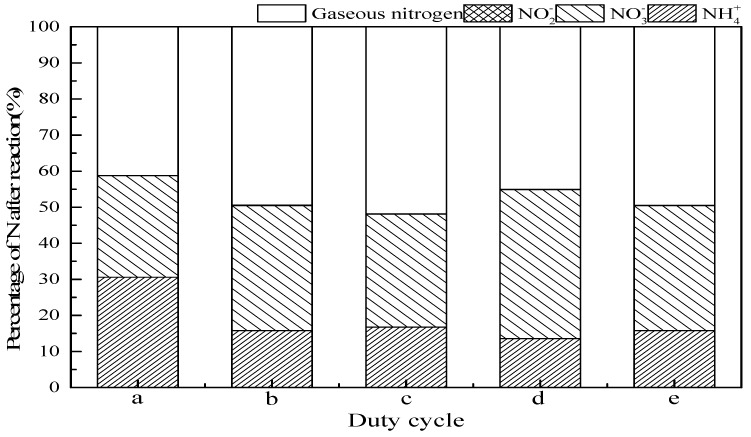
Effect of duty cycle on ammonia conversion. Reaction conditions: initial NH_4_^+^ concentration 50 mg/L, initial pH 9.5, ozone flow 0.8 g/h, catalytic dosage 2.0 g/L, temperature 25 °C, reaction time 60 min, ultrasonic frequency 25 kHz, ultrasonic power 270 W. (**a**) No ultrasound, (**b**) duty cycle 1:2, (**c**) duty cycle 1:3, (**d**) duty cycle 2:3, (**e**) duty cycle 2:4.

The US can be introduced either continuously or intermittently in the experiments. Since the continuous addition consumes a large amount energy, the intermittent method is adopted. To explore the effect of intermittent ultrasonic operation mode on ammonia conversion in the SrO-Al_2_O_3_/US/O_3_ process, five intermittent operation modes were set up, which were (a) no ultrasound, (b) ultrasonic operation 1 s and interval 1 s (duty cycle 1:2), (c) ultrasonic operation 1 s and interval 2 s (duty cycle 1:3), (d) ultrasonic operation 2 s and interval 1 s (duty cycle 2:3), and (e) ultrasonic operation 2 s and interval 2 s (duty cycle 2:4), as observed in Figure 8. When the duty cycle was 1:3, the ammonia conversion was 83.2%, gaseous nitrogen reached the maximum of 51.8% and the production of NO_3_^−^ 31.28%. When the duty cycle was 2:3, the ammonia conversion reached a maximum of 86.4%, gaseous nitrogen 41.3% and NO_3_^−^ production 45.0%. 

### 3.3. Effect of Tert-Butanol and Inorganic Ions on Ammonia Conversion 

#### 3.3.1. Effect of *Tert*-Butanol

**Figure 9:**
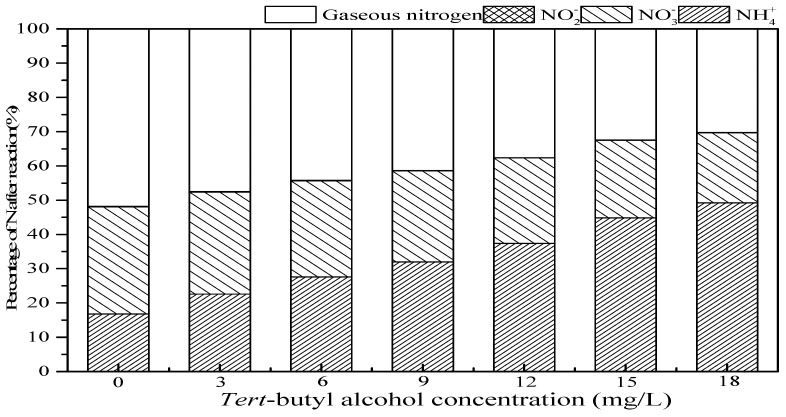
Effect of *tert*-butanol on ammonia conversion in SrO-Al_2_O_3_/US/O_3_ system. Reaction conditions: initial NH_4_^+^ concentration 50 mg/L, initial pH 9.5, ozone flow 0.8 g/h, catalytic dosage 2.0 g/L, temperature 25 °C, reaction time 60 min, ultrasonic frequency 25 kHz, ultrasonic power 270 W, duty cycle 1:3.

*Tert*-butanol, a common ·OH scavengers’ agent, is difficult to directly react with O_3_ (reaction rate constant is K = 3.0 × 10^−2^ M^−1^ S^−1^) but reacts quickly with ·OH (K = 6.0 × 10^8^ M^−1^ S^−1^) [38]. In addition, *tert*-butanol would react with ·OH in bulk solution to form inert substances, which inhibited the chain reaction of ·OH [39,40]. The effect of *tert*-butanol on ammonia conversion in the SrO-Al_2_O_3_/US/O_3_ system is shown in Figure 9. With *tert*-butanol concentration increasing from 0 to 18.0 mg/L, the residual NH_4_^+^ increased from 16.8% to 49.2%, and the percentage of NO_3_^−^ and gaseous nitrogen decreased from 31.3% to 20.5%, and 51.8% to 30.2%, respectively. That is to say, the addition of *tert*-butanol largely inhibits the ammonia oxidation in the SrO-Al_2_O_3_/US/O_3_ system, indicating that the intermediate product (·OH) plays an important role.

#### 3.3.2. Effect of Inorganic Ions

**Figure 10:**
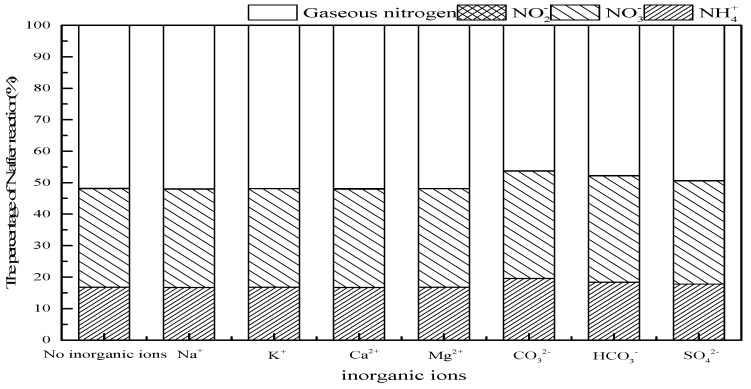
Effect of inorganic ions on ammonia conversion in SrO-Al_2_O_3_/US/O_3_ system. Reaction conditions: initial NH_4_^+^ concentration 50 mg/L, initial pH 9.5, ozone flow 0.8 g/h, catalytic dosage 2.0 g/L, temperature 25 °C, reaction time 60 min, ultrasonic frequency 25 kHz, ultrasonic power 270 W, duty cycle 1:3.

Large amounts of cations and anions often appear in the actual water body, the influence of main cations (Na^+^, K^+^, Ca^2+^, Mg^2+^) and anions (CO_3_^2−^, HCO_3_^−^, SO_4_^2−^) should be evaluated on ammonia conversion in SrO-Al_2_O_3_/US/O_3_ system. As observed in Figure 10, the addition of Na^+^, K^+^, Ca^2+^ and Mg^2+^ had no significant effect on ammonia conversion, while CO_3_^2−^, HCO_3_^−^ and SO_4_^2−^ suppressed the ammonia conversion. The anions’ radical-scavenging properties, as shown in Equations (13)–(15) [40,41,42], reduced the amount of ·OH produced, thereby causing a decrease in ammonia conversion.

(13) $${\mathrm{CO}}_{3}^{2-}+\cdot \mathrm{OH}\to {\mathrm{CO}}_{3}^{-}\cdot {+\mathrm{OH}}^{-}$$

(14) $${\mathrm{HCO}}_{3}^{-}+\cdot \mathrm{OH}\to {\mathrm{CO}}_{3}^{-}\cdot {+H}_{2}O$$

(15) $${\mathrm{SO}}_{4}^{2-}+\cdot \mathrm{OH}\to {\mathrm{SO}}_{4}^{-}\cdot {+\mathrm{OH}}^{-}$$

### 3.4. Actual Ammonium-Containing Wastewater Oxidized in the SrO-Al_2_O_3_/US/O_3_ System

The actual wastewater was collected from a rare earth metallurgical plant located in Longnan County, Jiangxi province, China and transferred into clean plastic bottles, and immediately transported to the laboratory for proper storage for future use. The characteristics of the actual wastewater used in this study are shown in Table 1. Actual wastewater with an initial NH_4_^+^ concentration of 50 mg/L was obtained by diluting the actual wastewater with an initial NH_4_^+^ concentration of 302 mg/L with deionized water. 

**Figure 11:**
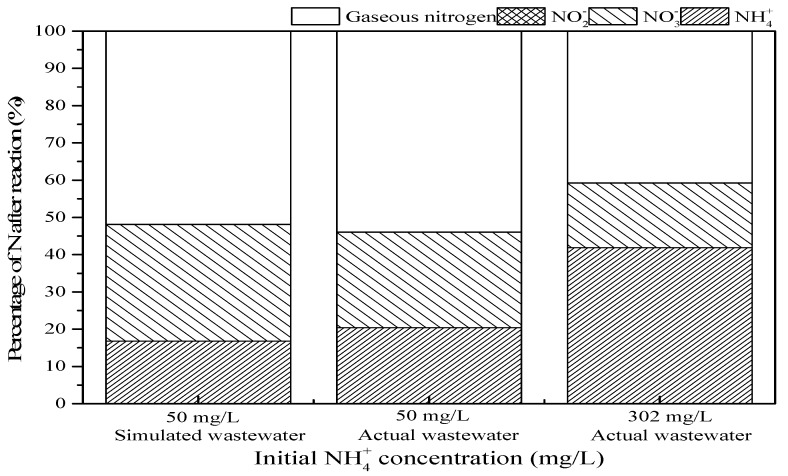
Comparison of oxidation of simulated wastewater and actual NH_4_^+^-containing wastewater in SrO-Al_2_O_3_/US/O_3_ system. Reaction conditions: initial pH 9.5, ozone flow 0.8 g/h, catalytic dosage 2.0 g/L, temperature 25 °C, reaction time 60 min, ultrasonic frequency 25 kHz, ultrasonic power 270 W, duty cycle 1:3.

As shown in Figure 11, there were some slight differences in the treatment between simulated water and actual wastewater with an initial NH_4_^+^ concentration of 50 mg/L. Among them, the ammonia conversion was 83.2% and 79.6%, respectively, and the gaseous nitrogen yield was 51.8% and 53.9%, respectively. However, when the actual wastewater with an initial NH_4_^+^ concentration of 302 mg/L was treated in SrO-Al_2_O_3_/US/O_3_ system, the ammonia conversion and gaseous nitrogen yield were 58.1% and 40.7%, respectively. This indicates that the treatment efficiency of the SrO-Al_2_O_3_/US/O_3_ process is limited and is currently only suitable for treating low concentrations of ammonia-containing wastewater.

### 3.5. Morphology Analysis of Catalyst

**Figure 12:**
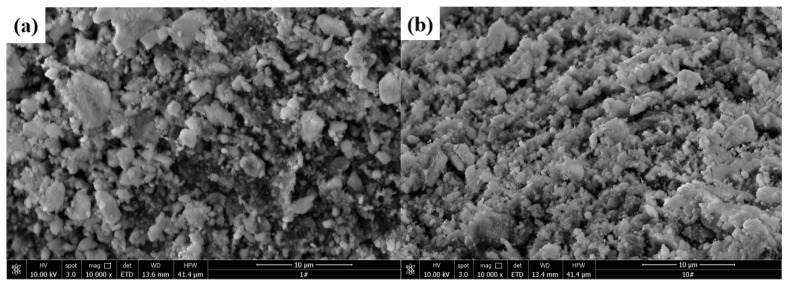
Morphology analysis of Al_2_O_3_ (**a**) and SrO-Al_2_O_3_ (**b**)

Surface topographies of Al_2_O_3_ and SrO-Al_2_O_3_ were observed by scanning electron microscopy (JSM-6360LV, Japanese electronics company, Japan) and the results are shown in Figure 12. Figure 12a shows that the surface of Al_2_O_3_ is rough and has obvious channels, which will facilitate the loading of SrO. Figure 12b shows that a large amount of irregular small particles is evenly distributed on the surface of SrO-Al_2_O_3_ and these small particles are closely joined.

### 3.6. The Pathway for Ammonia in Water Oxidized in the SrO-Al_2_O_3_/US/O_3_ System

**Figure 13:**
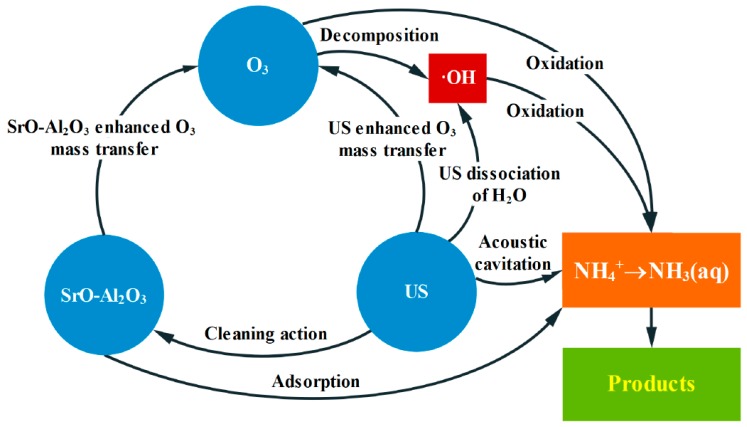
The pathway of ammonia in water oxidized by SrO-Al_2_O_3_/US/O_3_ system.

According to the above experimental results, the pathway for ammonia conversion in the SrO-Al_2_O_3_/US/O_3_ system can be inferred, as shown in Figure 13. Under alkaline conditions, ammonia mainly exists in a free state (NH_3_(aq)). The NH_3_(aq) oxidation occurs by both O_3_ and ·OH, whereas NH_4_^+^ oxidation is only carried out through ·OH. During ammonia conversion, the SrO-Al_2_O_3_ catalyst not only has a certain adsorption effect on NH_4_^+^, but accelerates the O_3_ decomposition to ·OH. US enhances the turbulence degree of liquid phase to promote the ammonia conversion to NH_3_(aq), while the cavitation effect of US causes H_2_O to crack to ·OH. In addition, US improves the mass transfer rate of O_3_, strengthens the O_3_ decomposition and produces more ·OH. Moreover, the ultrasonic wave cleans the surface of catalyst and slows down the deactivation of the catalyst, thus prolonging the service life of the catalyst. Therefore, the ammonia conversion has been greatly improved due to the synergistic effect of US, SrO-Al_2_O_3_ and O_3_ in the SrO-Al_2_O_3_/US/O_3_ system.

In this study, the products of oxidation of ammonia in aqueous solution are gaseous nitrogen in the gas phase and the residual NH_4_^+^, NO_2_^−^, and NO_3_^−^ in the liquid phase. Among them, gaseous nitrogen may include N_2_, N_2_O, NO, NO_2_, NH_3_ [13,43,44]. The production of NO_2_^−^ and NO_3_^−^ was derived from the oxidation of NH_4_^+^ and NH_3_ (aq).

## 4. Conclusions

The experimental results indicated that, compared with US, O_3_, US/O_3_, SrO-Al_2_O_3_/US and SrO-Al_2_O_3_/O_3_ systems, the highest ammonia conversion rate was obtained by the SrO-Al_2_O_3_/US/O_3_ system. The free ammonia (NH_3_ (aq)) was oxidized by both O_3_ and ·OH at high pH, while the NH_4_^+^ was oxidized through ·OH generated by the decomposition of O_3_ and H_2_O at low pH. Compared to the SrO-Al_2_O_3_/O_3_ system, the reaction time required in the SrO-Al_2_O_3_/US/O_3_ system is shortened, and the consumption of catalyst dosage and ozone was reduced. Moreover, reasonable control of ultrasonic power and duty cycle can further improve the ammonia conversion and gaseous nitrogen. Under the conditions of initial NH_4_^+^ concentration 50 mg/L, initial pH 9.5, ozone flow 0.8 g/h, catalytic dosage 2.0 g/L, temperature 25 °C, reaction time 60 min, ultrasonic frequency 25 kHz, ultrasonic power 270 W, and duty cycle 1:3, the ammonia conversion and gaseous nitrogen yield were 83.2% and 51.8%, respectively. The oxidation of ammonia in the SrO-Al_2_O_3_/US/O_3_ system was affected by *tert*-butanol, CO_3_^2−^, HCO_3_^−^, SO_4_^2−^, indicating that ·OH is an important oxidizer involved in the ammonia ozonation.

To better clarify the mechanism of oxidizing ammonia, it is necessary to further determine the components of gaseous nitrogen qualitatively and quantitatively.

## Figures and Tables

**Table 1:** Characteristics of actual wastewater.

Parameter	pH	NH_4_^+^	NO^3−^	NO_2_^−^	Mg	Si	Ca	Mn	Rb	Na	Sr	Y
Concentration (mg/L)	8.45	302	70.2	0.57	1.04	1.26	27.8	3.10	1.15	0.69	0.69	0.51
